# Supplementary material for: COVID-19 Vaccine Antibody Response in a Single-Center Urban Hemodialysis Unit
Source: Vaccines (Basel). 2023 Jul 18;11(7):1252. doi: 10.3390/vaccines11071252 (PMC10384404; doi:10.3390/vaccines11071252)
Supplement: Supplementary file 1 [file vaccines-11-01252-s001.zip › vaccines-2485467-supplementary.pdf]

Supplemental Material

Table S1. Baseline characteristics of the enrolled patients.

Characteristic		Total	COVID-19 Naïve N=22	Previous COVID-19 infection N=8	p- Value
Age (year)		61.2±2.6	60.5±3.3	63.1±3.7	0.66
Female Sex no. (%)		14 (46.7%)	8 (36.4%)	6 (75%)	0.06
African American race no. (%)		29 (96.7%)	21 (95.5%)	8 (100%)	0.54
Time on dialysis (year)		4.6±0.7	4.5±0.9	4.9±1.1	0.84
Comorbidities					
	Hypertension no. (%)	18 (60%)	13 (59.1%)	5 (62.5%)	0.87
	Diabetes no. (%)	16 (53%)	10 (45.5%)	6 (75%)	0.15
	Obesity no. (%)	1 (3.3%)	0 (0%)	1 (12.5%)	0.09
	Chronic heart failure no. (%)	3 (10%)	2 (9.1%)	1 (12.5%)	0.78
	COPD no. (%)	0	0	0	
	Cirrhosis no. (%)	0	0	0	
	On immune suppressive medicine	0	0	0	
	Autoimmune disease	0	0	0	
	Cancer	3 (10%)	3 (13.6%)	0 (0 %)	0.27
	Active smoking no. (%)	1 (3.3%)	1 (4.6%)	0	0.54
	Active Drug abuse no. (%)	0	0	0	
	Active Alcohol abuse no. (%)	0	0	0	
Types of vaccine					0.44
	BNT162b2mRNA	28 (93.3%)	21 (95.5%)	7 (87.5%)	
	mRNA-1273	2 (6.7%)	1 (4.5%)	1 (12.5%)	
Vaccine interval					
	Between the first and second dose (days)	32 ±5	36 ±7	23 ± 1	0.27
	Between the second and third dose (months)	6 ± 0.4	6 ± 0.4	7 ± 0.9	0.18

Table S2. Multiple logistic analysis comparing vaccine non-responders to responders in COVID-19 naïve group.

Risk factor		Adjusted OR	CI	p- Value
Age	Per year	1.2	0.77-1.80	0.46
Sex	Female	0.97	0.04-24.41	0.99
Time on Dialysis	Per year	1.34	0.74- 2.44	0.33
Hypertension	If yes	0.11	0.00-29.23	0.43
Diabetes	If yes	0.21	0.00-152.58	0.64
